# Average Consensus over Mobile Wireless Sensor Networks: Weight Matrix Guaranteeing Convergence without Reconfiguration of Edge Weights

**DOI:** 10.3390/s20133677

**Published:** 2020-06-30

**Authors:** Martin Kenyeres, Jozef Kenyeres

**Affiliations:** 1 Institute of Informatics, Slovak Academy of Sciences, Dúbravská Cesta 9, 845 07 Bratislava 45, Slovakia; 2 Sipwise GmbH, Europaring F15, 2345 Brunn am Gebirge, Austria; jkenyeres@sipwise.com

**Keywords:** average consensus, consensus algorithms, data aggregation, distributed algorithms, mobile computing, mobile wireless sensor networks, sensor fusion

## Abstract

Efficient data aggregation is crucial for mobile wireless sensor networks, as their resources are significantly constrained. Over recent years, the average consensus algorithm has found a wide application in this technology. In this paper, we present a weight matrix simplifying the average consensus algorithm over mobile wireless sensor networks, thereby prolonging the network lifetime as well as ensuring the proper operation of the algorithm. Our contribution results from the theorem stating how the Laplacian spectrum of an undirected simple finite graph changes in the case of adding an arbitrary edge into this graph. We identify that the mixing parameter of Best Constant weights of a complete finite graph with an arbitrary order ensures the convergence in time-varying topologies without any reconfiguration of the edge weights. The presented theorems and lemmas are verified over evolving graphs with various parameters, whereby it is demonstrated that our approach ensures the convergence of the average consensus algorithm over mobile wireless sensor networks in spite of no edge reconfiguration.

## 1. Introduction

### 1.1. Theothical Insight into Data Aggregation

**Figure 1:**
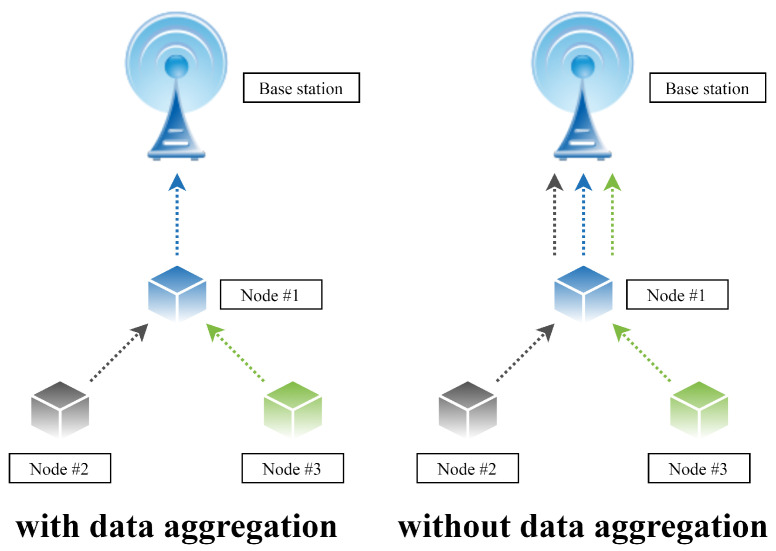
Comparison of wireless sensor network with and without data aggregation.

Over recent years, data (resp. information) have been becoming an increasingly valuable commodity in our technologically advanced society [1,2]. However, possessing an unorganized large amount of data can be counterproductive to its owner; therefore, the importance of data aggregation is gaining in importance nowadays [1,3,4]. It is because data aggregation can ensure gathering and expressing a typically large data amount in a summary form appropriate for further processing/analyzing. Thus, data aggregation is an essential process not only in technical industries (e.g., wireless sensor networks (WSNs), the Internet of Things (IoT), etc.), but also in other fields, such as the financial sector, investments, travel industry, etc. [1,2,3,4]. In many modern multi-agent systems, data aggregation mechanisms are applied to process independently measured data from multiple sources that are often deployed in extensive geographical areas [4,5,6]. Their application is indented to ensure sensor measurements with increased confidence, even though the precision of the sensor nodes is affected by many negative environmental factors (e.g., radiation, pressure variations, temperature, etc.) [7,8]. The goal of these mechanisms in multi-agent systems is to calculate/estimate an aggregate function (e.g., the arithmetic mean, the sum, the maximum/the minimum, etc.) from data measured by independent entities in order to create information that cannot be obtained by measurements executed by single sensor nodes, whereby the executed applications are optimized in many aspects [8]. In addition, mechanisms for data aggregation can eliminate highly-correlated or even duplicated data, thereby optimizing the overall energy consumption [8]. In Figure 1, we show an example of WSN with and with data aggregation.

According to [9], data aggregation mechanisms for multi-agent systems can be divided into two categories:

▪ Centralized data aggregation
▪ Distributed data aggregation

The first category, the centralized data aggregation, is based on the presence of a data fusion center in a system. Its goal is to collect data that are measured by the other entities in the system via wireless communication. The measured data can be transmitted to a data fusion center in two ways—either directly or by a multi-hop relay. A multi-hop relay is not an optimal way for systems with mobile entities, since it requires the implementation of routing mechanisms. Therefore, the other category, the distributed data aggregation, has gained in importance and frequently substituted the less sophistical centralized approach over recent years. These approaches are built-up on the absence of data fusion centers and any global information about the system. Their principle lies in neighbor-to-neighbor communication and local updates at each entity in a system. Eventually, the entities are supposed to have the exact value of the estimated aggregate function or at least its precise estimate.

As literature review shows [3,10,11,12,13,14], distributed consensus-based algorithms have found application as mechanisms for data aggregation in a wide range of disciplines, such as WSNs, cloud computing, IoT, blockchains, etc.

### 1.2. Consensus Theory

A consensus (agreement) problem, a subcategory of computer science, poses one of the most fundamental problems in numerous multi-agent systems and, therefore, has attracted significant attention of many scientists over the past years [15,16]. A consensus means that a set of the spatially-distributed independent entities with a scalar/vector initial values reach an agreement on a particular quantity (e.g., the computation of the average temperature value in (wireless) sensor networks, aiming at the same moving direction in robotic systems, etc. [16]) without any central coordination and any global communication [16,17]). The entities are connected to each other through potentially time-variant networks and they are only aware of their neighbors, with which they communicate [16]. According to this communication and local update rules, the entities are able to reach an agreement on a particular value [16].

**Figure 2:**
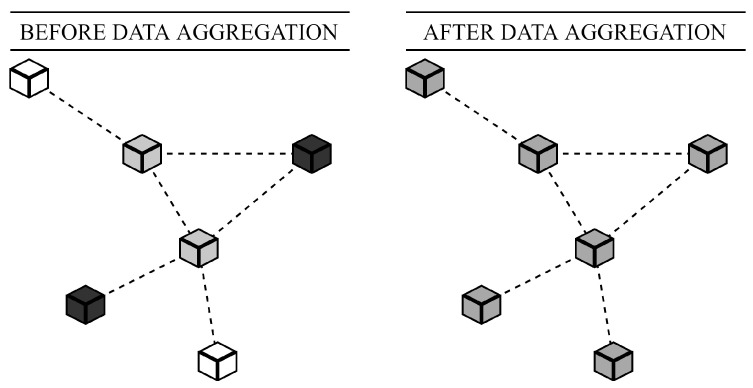
Graphical demonstration of AC for distributed averaging over multi-agent systems with six entities (shade of grey represents value of inner state)—right figure represents that entities are in agreement.

From the family of consensus-based algorithms, we focus our attention on average consensus (AC)—a distributed multi-functional algorithm finding the application in various areas. Over the past decades, AC has been significantly studied in computer science and related fields [18]. In this paper, we address AC for data aggregation over mobile multi-agent systems, or more specifically, AC for distributed averaging. However, due to its high versatility, AC can be applied to estimating also other aggregate functions—the sum and the graph order (The graph order *n* is the number of the vertices in a graph.) [19]. AC enables the inner states of all the entities in a system to asymptotically converge to the arithmetic mean determined by all of the initial inner states in an iterative fashion [18]. Eventually, each entity knows an estimate of the wanted aggregate function. In Figure 2, we graphically demonstrate the objective of AC on a multi-agent system formed by six entities. The color of the entities (or more specifically, the shade of the grey) represents the value of their initial inner state (a not specified measured value). The shade of the gray in the right figure is calculated as the average from these three colors (each component of the RGB scheme is separately averaged).

### 1.3. Mobile Wireless Sensor Networks

**Figure 3:**
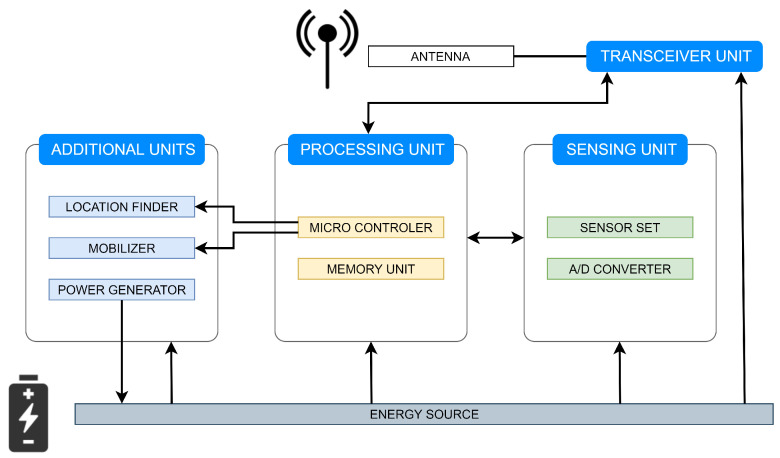
Architecture of mobile sensor node.

Mobile wireless sensor networks (MWSNs), a subclass of WSNs, are self-configuring and self-healing systems that consist of numerous mobile entities connected to each other via a wireless medium [20]. As their name evokes, mobility plays a crucial role in the execution of the MWSN-based applications [21]. The mobility of sensor/sink nodes in MWSNs can be achieved in different manners, e.g., equipping them with mobilizers for controlling their geographical location, fastening them to moving objects (e.g., vehicles, animals, peoples, robots, drones, etc.), etc. [22,23]. As stated in [20], it depends on the application requirements whether sensor nodes or sink nodes (possibly both) can change their position over time (either dependently/independently of each other) after the initial deployment. The sensor nodes in these systems are versatile devices and, therefore, can sense numerous various physical quantities (e.g, temperature, light, pollution, seismic events, air pressure, motion, humidity, wind, etc.); therefore, MWSNs find application in many areas, such as healthcare monitoring, traffic monitoring, social interaction, security systems, etc. [24,25,26]. As shown in Figure 3, the sensor nodes in MWSNs consists of three main components (namely, the sensing unit, the processing unit, and the transceiver unit), several additional units, and the energy source [24]. The sensing unit is responsible for sensing physical quantities of interest from the surrounding environment and converting the measured information into a digital form [24]. The goal of the processing unit is to process all the data and control the operation of the other components [24]. The transceiver unit enables transmitting and receiving data over the adjacent geographical area [24]. All of these components can operate due to the power supply provided by the energy source [24]. The additional units, such as the location finder, the mobilizer, and the power generator, allow the sensor nodes to change their geographical location and recharge the energy source, respectively [24]. When compared to static WSNs, MWSNs find a wider variety of applications, since the mobility of entities results in many advantages, such as reliability, cost, improved coverage, connectivity, energy efficiency, dynamic topology, increased channel capacity, etc. [26,27,28]. Moreover, MWSNs can easily monitor also moving targets (e.g., animals, people, chemical clouds, etc.) [29]. However, mobility may cause several issues, e.g., reliable data transfer, contact detection, mobility control, mobility-aware power management, latency problems, etc. [26], and thus, reaching a consensus in the mobile systems is more complicated than in their static variant [30].

### 1.4. Our Contribution

Our contribution that is presented in this paper is motivated by an effort to struggle with constraints affecting the execution of AC over MWSNs. In many WSN-based applications, the sensor nodes suffer from limited computation, communication, and energy capabilities; therefore, numerous research activities have been focused on optimization of the algorithms for WSNs. This motivates us to propose an approach that simplifies the execution of AC over MWSNs in the aspects that are mentioned above. First, the presented approach is proposed in such a way that communication among the sensor node is decreased (i.e., during the algorithm execution, it is not necessary to track parameters, such as the maximum degree, the Laplacian eigenvalues, the degrees of the neighbors, etc.—and so, the parameters ensuring the proper operation of AC). Subsequently, it is not necessary to reconfigure these parameters during the algorithm execution, whereby computation requirements are optimized (e.g., other complementary algorithms for determining parameters such as the maximum degree, the Laplacian eigenvalues, etc. do not have to be implemented). The optimization of the mentioned aspects results in decreased energy requirements, thereby prolonging the life-time of MWSN-based applications. Moreover, it is often a difficult task (especially, in fast-changing topologies) that all of the nodes achieve agreements on the same value of the parameters such as the maximum degree, the Laplacian spectrum, etc. (so, the value of any of these parameters may change before each sensor node can determine its value). Note that incorrectly determined algorithm parameters may result in the collapse of the whole system.

It is a well-known fact that AC operates properly, provided that three convergence conditions are met. Our contribution consists of a mathematical derivation of the upper bound of the mixing parameter (The mixing parameter for weighting states received from the adjacent area.) guaranteeing the meeting of the third convergence condition of AC over MWSNs without the need to reconfigure the mixing parameter during the algorithm execution. Subsequently, we provide how to meet the two remaining convergence conditions, i.e., we determine the weights for the inner state at each sensor node for the next iteration. Finally, we compose a weight matrix simplifying the execution of AC over MWSNs.

More specifically, we mathematically prove that the optimal uniform edge weights (referred to as the Best Constant edge weights) of a complete finite graph (A complete graph is an undirected simple graph where each vertex is linked to all the others.) with an arbitrary order ensures that the third convergence condition is met in all of its spanning subgraphs (A spanning subgraph is a subgraph of a graph such that the vertex set does not change; meanwhile, the edge set may vary.); therefore, it is sufficient for the convergence achievement at each iteration over MWSNs to configure the edge weights at the beginning of the algorithm to the mixing parameter of Best Constant weights of the complete finite graph of the corresponding order and to recalculate the “self-loop” weights (The so-called self-loop weights are considered to be the weights multiplying the inner states at the corresponding sensor nodes, i.e., the diagonal entries of the weight matrix.) at each iteration. Because of this, the convergence is ensured in each undirected simple finite graph (An undirected simple finite graph is a graph without any loops and any multiple edges, with a finite number of both the vertices and the edges, and all the edges are bidirectional.) forming a graph sequence representing MWSN, whereby AC operates correctly over mobile systems in spite of no edge reconfiguration. Thus, each entity in a mobile multi-agent system only has to track its degree (The degree of a node is the number of its neighbors.) and updates its “self-loop” weight whereby only the diagonal elements of the weight matrix (The weight matrix determines the weights of the inner states. Its entries are determined by the applied consensus algorithm, see Section 3.2 for further details.) are recofigured during the algorithm execution. Thus, no information, such as the maximum degree, the Laplacian spectrum, etc., about the graphs forming a graph sequence is required to be known in contrast to related contributions. Accordingly, our approach significantly simplifies AC over mobile systems in terms of the computation and communication requirements. In the literature, one can find several papers concerning with AC over mobile systems (see Section 2). However, these approaches are based on a significant recalculation (Many of them require additional knowledge such as the maximum degree, the Laplacian spectrum, etc. which may be hard to determine in time-varying systems.) of the weight matrix during the algorithm execution; therefore, they are not as optimal for real-world implementation as our contribution. Thus, we do not compare our contribution to related work in terms of performance optimization, as our goal is to simplify AC, which is not possible to be numerically quantified.

### 1.5. Paper Organization

The next section of this paper (Section 2) is concerned with papers that address AC over mobile multi-agent systems. In that section, we compare the novelty of our paper to the presented related papers. Section 3 is divided into two subsections. The first subsection, Section 3.1, deals with the applied mathematical tools for modeling MWSNs. In the other subsection (Section 3.2), the definition of AC over mobile systems and its convergence conditions are provided. Section 4 is divided into three separate subsections. In the first one (Section 4.1), we describe the Laplacian spectrum of complete finite graphs and their spanning subgraphs. The second subsection (Section 4.2) consists of our contribution—a derivation of the upper bound of the mixing parameter guaranteeing the convergence over MWSNs without any reconfiguration of edge weights, a subsequent recalculation of the edge weights, and finally, the design of a weight matrix simplifying AC over MWSNs. In the last one (Section 4.3), we focus our attention on critical topologies and prove that the proposed weight matrix also ensures the convergence in undirected simple finite disconnected, undirected simple finite bipartite regular, and undirected simple finite disconnected graphs with bipartite regular components. Section 5 contains two subsections. In the first one (Section 5.1), the applied research methodology is introduced. In the other subsection (Section 5.2), we provide and discuss the results from numerical evaluations carried out in Matlab2018a. Section 6 briefly summarizes our contribution that is presented in this paper.

## 2. Related work

In this section, we deal with papers that are concerned with a consensus problem over systems with time-variant topologies and compare our contribution to the following papers. According to the primary purpose of the related papers, we divide them into three categories:

▪ Section 2.1: Contributions addressing a positive impact of mobility on performance
▪ Section 2.2: Contributions addressing the convergence achievement in disconnected topologies
▪ Section 2.3: Other contributions

### 2.1. Contributions Addressing a Positive Impact of Mobility on Performance

In [31], the authors exploit a mobile node in a network for fast discrete-time AC. They propose a protocol that is based on networks formed by both static nodes and the mobile node and show that mobility can be used to accelerate the convergence rate. They define the time-variant weighting parameter, which can take two states (“0” or “1”) and affects the weight matrix and the inner state of the mobile node. In this approach, the mobile node does not execute the algorithm, but is for transmitting its inner state to other nodes, which are static. At most one static node can communicate during each communication. The authors of [30] analyze the mean square error (MSE) of AC executed over MWSNs modeled as stationary evolving random geometric graphs. According to the presented results from numerical evaluations, the authors state that mobility improves the performance of AC (e.g., the algorithm is accelerated, the energy required for transmission is reduced, etc.). Moreover, the authors identify that meeting the convergence conditions at each iteration is sufficient for ensuring the algorithm convergence (i.e., the weight matrix has to meet the convergence conditions at each iteration). In this approach, the weight matrices are reconfigured during the algorithm execution according to locally tracking the current degree of each node. The authors of [32] deal with a consensus-based estimation over relay assisted sensor networks that are proposed for situation monitoring, where relay nodes with a varying position are used for data aggregation. The update rule of the sensor nodes is determined by (among others) the time-variant decaying weight. In [33], it is identified that the max/min consensus algorithm for extrema finding achieve, in general, higher performance than in static graphs. Additionally, it is shown that an increase in the number of the mobile nodes optimizes both the estimation precision and the algorithm rate. The highest performance is observed in the case that all of the entities are mobile.

### 2.2. Contributions Addressing the Convergence Achievement in Disconnected Topologies

In [17], the authors focus their attention on several aspects of AC over mobile networks, namely time-varying signals, the robustness to arbitrary non-uniform time delays, and disconnected topologies. The authors prove that the value to which the inner states converge is preserved, even though networks are split and then merged again; therefore, a temporary partition of networks does not affect the value of the aggregate function to which the inner states converge. The authors of [34] propose an algorithm ensuring that the average consensus problem can be solved, despite switching topologies in the case when the networks switch between instantaneously balanced, connected-over-time networks. It means that the consensus is asymptotically obtained when a network topology is balanced at each iteration, and the union of the graphs is strongly connected over each interval T. Moreover, the authors examine the “deadbeat” consensus, which means that the consensus is obtained in a finite time by applying a message-passing mechanism. In [35], the authors deal with a consensus problem over multi-agent systems with limited unreliable information transfers and dynamic topologies. They propose discrete/continuous update rules and show that the consensus can be obtained in directed graphs if the graph union has a spanning tree. It is a significantly less strict requirement than the one from [36], where it is stated that the union of graphs has to be connected frequently enough to achieve the consensus among moving nodes (however, the condition from [36] is valid also for undirect graphs). The update rule in [35] is based on the weighting parameter that is time-variant and has to be greater than zero. Additionally, in [37], it is concluded that the convergence of consensus-based generalized Metropolis-Hastings algorithm is guaranteed if graphs are connected in the long term. Moreover, they identify that a decrease in the mixing parameter results in performance optimization of the mentioned algorithm over mobile systems.

### 2.3. Other Contributions

The paper [38] is concerned with the non-linear consensus filtering problem with observations that are intermittent over mobile networks and the proposal of an algorithm that is based on combining the cubature information filtering with consensus-based algorithms. In this paper, AC is enhanced within the framework of the cubature Kalman filtering algorithm in the information form. Every node obtains the information state contribution and the correlation information and adds this information in the predicted information state vector and the information matrix. This ensures an improved result of the implemented filter. In [39], the authors state that it is required to know an upper bound on the degrees for the convergence achievement in time-varying systems. Moreover, they also analyze the Metropolis update (respectively, its so-called lazy version), i.e., each node has to be aware of time-variant degrees of its neighbors.

When compared to the presented papers, the main idea of our contribution lies in a simplification of AC over mobile networks, i.e., we propose an algorithm that does not require reconfiguration of the edge weights during its execution. This is assumed to significantly simplify the algorithm in terms of the computation and the communication requirements.

## 3. Problem Formulation: Average Consensus over Mobile Wireless Sensor Networks

### 3.1. Mathematical Model of Mobile Wireless Sensor Networks

In this paper, the model of MWSNs formed by *n* sensor nodes consists of two parts: the initial graph and the evolving graph (Note that some sources consider the initial graph to be a part of an evolving graph.) [40,41,42]. Evolving graphs, probably the most general way to describe time-variant networks, can be defined as an infinite sequence of graphs {*G*${}_{1}$, *G*${}_{2}$, …} on the same vertex set **V** (and also on the same vertex set as the initial graph) [41,42]. In this paper, we apply two models of evolving graphs, namely stationary Markovian evolving graphs (labeled as SMEGs) and stationary edge-Markovian evolving graphs (SEMEGs). Note that there are also other definitions of evolving graphs in the literature [43,44,45].

The first model, SMEGs, is defined as a sequence of stochastic graphs with the Markov property forming a Markov chain [41]. Having the Markov property means that the present graph is independent of the previous and the future graphs that form the corresponding Markov chain [41]. In our analyses, this model is determined by the probability *p*${}_{ef}$, which conditions the existence of an edge in a graph forming the Markov chain. In the presented model, its value is the same for each graph edge and it does not vary for different graphs. So, an edge between two arbitrary vertices exists with the probability *p*${}_{ef}$ in a graph.

The other applied model is SEMEGs, where the existence/absence of any graph edge in a graph from a Markov chain is conditioned by its existence/absence in the previous graph from the corresponding Markov chain [42]. The existence/absence of any graph edge is determined according to a two-state Markovian process with two probabilities, namely the birth-rate *p* and the death-rate *q* [42]. Thus, an edge exists in a graph *G*${}_{k}$ (Here, *k* is the label of an iteration.) with the probability equal to the birth-rate *p* and does not exist with the probability 1 −*p* in the case of absenting in *G*${}_{k-1}$. Analogically, it does not exist in *G*${}_{k}$ with the probability *q* and exists with the probability 1 −*q*, provided that this edge is present in *G*${}_{k-1}$. This procedure can be described by the transition matrix **M**, where the existence of an edge in a graph is represented by “1” and its absence by “0”, as follows [42]:

(1) M=00101−pp1q1−q

By definition, the initial graph and each graph from a Markov chain (regardless of the type of evolving graphs) are determined by the vertex set **V** (This set is the same for each graph from a Markov chain, including the initial graph *G*${}_{0}$.) formed by all of the graph vertices representing the sensor nodes in MWSN, i.e., **V** = {*v*${}_{1}$, *v*${}_{2}$, …, *v*${}_{n}$}, and the edge set **E** (This set is very likely to be different for various graphs from a Markov chain.), which contains all of the graph edges indicating a one-hop connection between two vertices (an edge *e*${}_{ij}$ links *v*${}_{i}$ and *v*${}_{j}$) [30]. In MWSNs, an arbitrary sensor node is able to directly receive a message from a message-sending sensor node in the case of being covered by the transmission range of the sender. The ability to receive a message from a particular sensor node is indicated by the existence of an edge.

**Figure 4:**
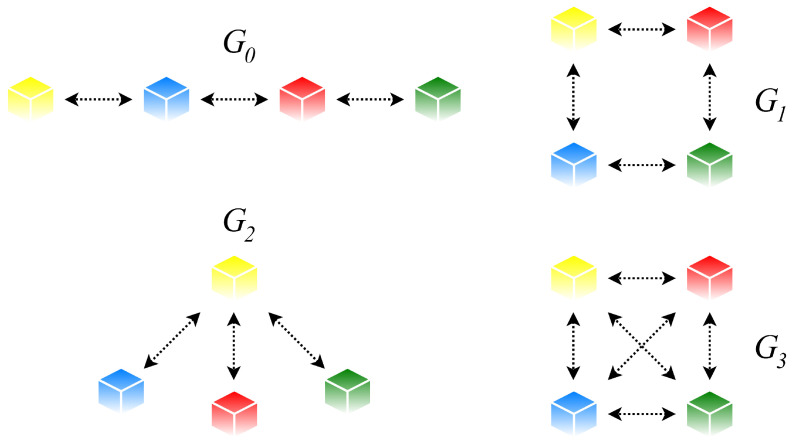
Example of evolving graph (including the initial graph) with order *n* = 4.

Thus, MWSNs are represented by the undirected simple finite initial graph *G*${}_{0}$, which has the stationary distribution of the corresponding Markov chain, and a sequence of undirected simple finite graphs forming a stationary Markov chain, i.e., (*G${}_{k}$*)${}_{k\geq 0}$ [41,42]. Note that matrices describing the graphs forming a Markov chain (and also the initial graph) can have a different zero pattern (The zero pattern of a matrix is a (0,1)-matrix obtained from this matrix in such a way that each non-zero entry (or possibly non-zero) takes the value one and, analogically, each zero entry is equal to zero.), complicating reconfiguration of the weight matrix during the algorithm execution [30]. In Figure 4, we show an example of a graph sequence forming an evolving graph (including the initial graph).

The applied mathematical models are designated to model networks with the constant number of the sensor nodes. Therefore, they cannot reflect the scenarios where sensor nodes can be removed/added from/in the network. Additionally, we assume undirected graphs, i.e., the connection between two vertices is always mutual, preventing modeling scenarios where the sensor nodes are heterogenous in the transmission range. Accordingly, for modeling the mentioned scenarios, more appropriate models can be found in the literature [43,44,45].

Now, let us turn our attention to how to describe the graph topology. One of the most common ways is the Laplacian matrix defined, as follows [46]:

(2) [L(G)]ij=−1,if eij∈Edi,if i=j−0,otherwise

Here, *d*${}_{i}$ is the degree of *v*${}_{i}$. Subsequently, we can define the Laplacian spectrum of the corresponding graph as follows [47]:

(3) $$Spec\left(L\left(G\right)\right)=\left\{\lambda_{1}\left(G\right),\lambda_{2}\left(G\right),\ldots ,\lambda_{n}\left(G\right)\right\}$$

The eigenvalues of the Laplacian spectrum are sorted in the non-increasing order, i.e., $\lambda_{1}$(*G*) ≥$\lambda_{2}$(*G*) ≥ … ≥$\lambda_{n-1}$(*G*) ≥$\lambda_{n}$(*G*) [47].

### 3.2. Average Consensus over Mobile Systems

As mentioned earlier, AC is a distributed multi-functional algorithm for data aggregation—we focus on distributed averaging in this paper. Thus, all of the inner states asymptotically converge to the arithmetic mean in an iterative fashion. Initially, each entity *v*${}_{i}$∈**V** is allocated the inner state represented by a scalar value *x*${}_{i}$(0) (Labeling *k* = 0 represents the initial inner states.) (all of the inner states at the corresponding iteration are gathered in **x**(*k*)) [48], which is updated at each iteration according to the current inner state and the inner states collected from the neighbors, as follows [30]:

(4) x(k+1)=W(k)x(k)

Here, **W**(*k*) is the weight matrix variant over the iterations (The weight matrix **W** is a function of an iteration, since a mobile system is very likely to be described by a different graph at different iterations.), whose elements affect several aspects, e.g., the convergence/divergence of the algorithm, the convergence rate, the robustness, etc. Our contribution results from the Perron matrix (It means that all the edges take the same weight.), which is defined as follows [49]:

(5) W=I−ϵL(G)

Here, ϵ is the mixing parameter, and **I** is the identity matrix [49]. As stated in [30], AC operates correctly over mobile systems, provided that these three convergence conditions (we refer to (6) as the first convergence condition, (7) as the second convergence condition, and (8) as the third one) are met at each iteration *k* (Note that we provide all three convergence conditions in contrast to [30], where only two conditions are provided.):

(6) $$1^{T}W\left(k\right)=1^{T}$$

(7) W(k)1=1

(8) $$\rho \left(W\left(k\right)-\frac{1}{n}11^{T}\right)<1$$

Here, **1** is a column all-ones vector, **1**${}^{T}$ is its transpose (The transposed matrix is the flipped variant of the original matrix.), and ρ(·) is the spectral radius of the analyzed vector/matrix defined, as follows [30,50]:

(9) $$\rho \left(\cdot \right)=\underset{i}{max}\left\{|\lambda_{i}\left(\cdot \right)|:i=1,2,\ldots n\right\}$$

## 4. Design of Weight Matrix Simplifying Average Consensus Algorithm over Mobile Wireless Sensor Networks

In this section, we identify how to meet all three convergence conditions that are provided in (6)–(8) in each undirected simple finite graph on *n* vertices from the corresponding Markov chain (In the following paragraphs, we talk also about the initial graph *G*${}_{0}$ when referring to a Markov chain.) without any reconfiguration of the edge weights. Accordingly, in our contribution, the edge weights are configured in the initial graph *G*${}_{0}$ and kept unchanged during the algorithm execution. Thus, only the “self-loop” weights (i.e., the diagonal elements of the weight matrix) have to be recalculated. In the first subsection (Section 4.1), we identify how the eigenvalues of a complete finite graph with an arbitrary order *n* and the eigenvalues of its arbitrary spanning subgraph interlace. Based on these findings, we compose a weight matrix guaranteeing the convergence of AC over MWSNs without any reconfiguration of the edge weights in the second subsection (Section 4.2). The third subsection (Section 4.3) is concerned with a convergence analysis of the designed weight matrix in critical topologies, namely in undirected simple finite bipartite regular graphs, in undirected simple finite disconnected graphs, and in undirected simple finite disconnected graphs with bipartite regular component(s).

### 4.1. Laplacian Spectrum of Complete Finite Graphs and Their Spanning Subgraphs

At first, let us focus on Theorem 1.1 from [47], on which our contribution is mainly based, and reformulate it, as follows:

**Theorem** **1.** *Let G be an undirected simple finite non-empty graph on n vertices, and let H = G − e${}_{ab}$ be its spanning subgraph obtained from G by removing an arbitrary edge e${}_{ab}$ from G. Subsequently, the eigenvalues of these two graphs interlace, as follows:*

(10) $$0=\lambda_{n}\left(H\right)=\lambda_{n}\left(G\right)\leq \lambda_{n-1}\left(H\right)\leq \lambda_{n-1}\left(G\right)\leq \ldots \leq \lambda_{2}\left(H\right)\leq \lambda_{2}\left(G\right)\leq \lambda_{1}\left(H\right)\leq \lambda_{1}\left(G\right)$$

*Additionally, so:*

(11) $$\lambda_{i}\left(H\right)\leq \lambda_{i}\left(G\right),\;\mathrm{for}\;i\;=\;1,2,\;\ldots \;n$$

**Proof of Theorem** **1.** An analogy with Theorem 1.1 from [47], a proof omitted. See [51] for a proof of Theorem 1.1. □

The following theorem is the most fundamental part of our contribution. Based on Theorem 1, we mathematically derive how the eigenvalues of the complete finite graph *K*${}_{n}$ on *n* vertices and the eigenvalues of all its spanning subgraphs interlace.

**Theorem** **2.** *Let $K_{n}$ be the complete finite graph on n vertices and with $\frac{n(n-1)}{2}$ edges, and let H be a spanning subgraph of $K_{n}$ obtained by removing an arbitrary number of edges from $K_{n}$, i.e., H = $K_{n}$− {e${}_{ab}$, …}, and **E** (H) = **E** ($K_{n}$)\{e${}_{ab}$, …}. Afterwards, the largest and second smallest eigenvalues of these two graphs interlace, as follows:*

(12) $$\lambda_{1}\left(H\right)\leq \lambda_{1}\left(K_{n}\right)$$

(13) $$\lambda_{n-1}\left(H\right)\leq \lambda_{n-1}\left(K_{n}\right)$$

**Proof of Theorem** **2.** According to Theorem 1, removal of one arbitrary edge *e*${}_{ab}$ from *K*${}_{n}$ causes that the eigenvalues of *K*${}_{n}$ and its spanning subgraph *H*${}_{1}$ = *K*${}_{n}$−*e*${}_{ab}$ (**E**(*H*${}_{1}$) = **E**(*K*${}_{n}$)\{*e*${}_{ab}$}) (The index of *H* represents the number of the removed edges from *K*${}_{n}$.) interlace as follows:

(14) $$0=\lambda_{n}\left(H_{1}\right)=\lambda_{n}\left(K_{n}\right)\leq \lambda_{n-1}\left(H_{1}\right)\leq \lambda_{n-1}\left(K_{n}\right)\leq \ldots \leq \lambda_{2}\left(H_{1}\right)\leq \lambda_{2}\left(K_{n}\right)\leq \lambda_{1}\left(H_{1}\right)\leq \lambda_{1}\left(K_{n}\right)$$

Next, we remove a further edge *e*${}_{cd}$ now from *H*${}_{1}$ and obtain a spanning subgraph *H*${}_{2}$ = *H*${}_{1}$−*e*${}_{cd}$ = *K*${}_{n}$− {*e*${}_{ab}$, *e*${}_{cd}$} (**E**(*H*${}_{2}$) = **E**(*H*${}_{1}$)\{*e*${}_{cd}$} = **E**(*K*${}_{n}$)\{*e*${}_{ab}$, *e*${}_{cd}$}). Then, the eigenvalues of *H*${}_{1}$ and *H*${}_{2}$ interlace as follows:

(15) $$0=\lambda_{n}\left(H_{2}\right)=\lambda_{n}\left(H_{1}\right)\leq \lambda_{n-1}\left(H_{2}\right)\leq \lambda_{n-1}\left(H_{1}\right)\leq \ldots \leq \lambda_{2}\left(H_{2}\right)\leq \lambda_{2}\left(H_{1}\right)\leq \lambda_{1}\left(H_{2}\right)\leq \lambda_{1}\left(H_{1}\right)$$

This procedure can be repeated until all of the edges of *K*${}_{n}$ are removed. Thus, the eigenvalues of *H*${}_{\frac{n(n-1)}{2}-1}$ (an undirected simple finite graph whose size is equal to one) and the eigenvalues of the empty graph *H*${}_{\frac{n(n-1)}{2}}$ on *n* vertices interlace, as follows:

(16) $$\begin{array}{c} 0=\lambda_{n}\left(H_{\frac{n(n-1)}{2}}\right)=\lambda_{n}\left(H_{\frac{n(n-1)}{2}-1}\right)\leq \lambda_{n-1}\left(H_{\frac{n(n-1)}{2}}\right)\leq \lambda_{n-1}\left(H_{\frac{n(n-1)}{2}-1}\right)\leq \ldots \\ \ldots \leq \lambda_{2}\left(H_{\frac{n(n-1)}{2}}\right)\leq \lambda_{2}\left(H_{\frac{n(n-1)}{2}-1}\right)\leq \lambda_{1}\left(H_{\frac{n(n-1)}{2}}\right)\leq \lambda_{1}\left(H_{\frac{n(n-1)}{2}-1}\right) \end{array}$$

According to the above-mentioned dependencies, we can bound the largest eigenvalues, as follows:

(17) $$\lambda_{1}\left(H_{\frac{n(n-1)}{2}}\right)\leq \lambda_{1}\left(H_{\frac{n(n-1)}{2}-1}\right)\leq \ldots \leq \lambda_{1}\left(H_{2}\right)\leq \lambda_{1}\left(H_{1}\right)\leq \lambda_{1}\left(K_{n}\right)\Rightarrow \lambda_{1}\left(H\right)\leq \lambda_{1}\left(K_{n}\right)$$

Analogically, for the second smallest eigenvalue, we can state the following:

(18) $$\begin{array}{c} \lambda_{n-1}\left(H_{\frac{n(n-1)}{2}}\right)\leq \lambda_{n-1}\left(H_{\frac{n(n-1)}{2}-1}\right)\leq \ldots \leq \lambda_{n-1}\left(H_{2}\right)\leq \lambda_{n-1}\left(H_{1}\right)\leq \lambda_{n-1}\left(K_{n}\right)\Rightarrow \\ \lambda_{n-1}\left(H\right)\leq \lambda_{n-1}\left(K_{n}\right) \end{array}$$

Note that *K*${}_{n}$ is the supergraph of each *H* on *n* vertices. □

**Figure 5:**
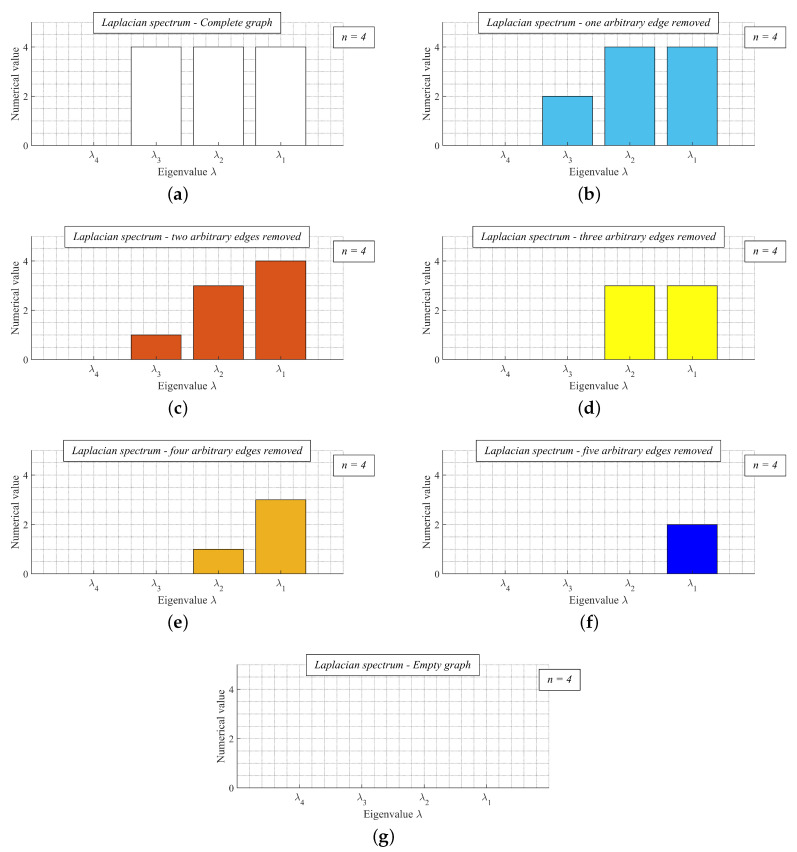
Laplacian spectrum of complete graph with order *n* = 4 and laplacian spectrum of its six arbitrary spanning subgraphs including empty graph. (**a**) Complete finite graph *K*${}_{4}$; (**b**) Arbitrary subgraph *H*${}_{1}$ obtained by removing one arbitrary edge from *K*${}_{4}$; (**c**) Arbitrary subgraph *H*${}_{2}$ obtained by removing one arbitrary edge from *H*${}_{1}$; (**d**) Arbitrary subgraph *H*${}_{3}$ obtained by removing one arbitrary edge from *H*${}_{2}$; (**e**) Arbitrary subgraph *H*${}_{4}$ obtained by removing one arbitrary edge from *H*${}_{3}$; (**f**) Arbitrary subgraph *H*${}_{5}$ obtained by removing one arbitrary edge from *H*${}_{4}$; (**g**) Empty graph *H*${}_{6}$ obtained by removing one arbitrary edge from *H*${}_{5}$.

**Figure 6:**
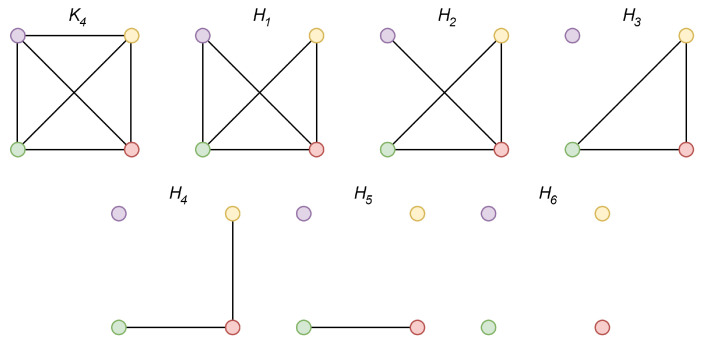
Topologies of graphs whose spectrum is depicted in Figure 5.

In Figure 5, we provide a valid example of Theorem 2—the Laplacian spectrum of the complete finite graph with the order *n* = 4 and its six arbitrary spanning subgraphs. We can see from the figures that the largest Laplacian eigenvalue of the complete graph $\lambda_{1}\left(K_{4}\right)$ is greater than/or equal to $\lambda_{1}$ of its every spanning subgraph (compare Figure 5a with Figure 5b–g), and $\lambda_{3}\left(K_{4}\right)$ is greater than/or equal to $\lambda_{3}$ of its every spanning subgraph. Thus, it is seen from the presented results that the expressions (12) and (13) are valid. In Figure 6, we show the graphs whose spectrum is depicted in Figure 5.

### 4.2. Weight Matrix Guaranteeing Convergence over Mobile Wireless Sensor Networks without Reconfiguration of Edge Weights

In the following part, we compose a weight matrix simplifying AC over MWSNs based on Theorem 2. As mentioned earlier, the convergence of AC is obtained, provided that the three convergence conditions (6)–(8) are met. At first, we derive the upper bound of the mixing parameter ϵ, guaranteeing that the convergence condition (8) is met in each graph forming a Markov chain. Subsequently, we provide how to recalculate “self-loop” weights so that the convergence conditions (6) and (7) are met. Our contribution is mainly based on the finding that the Best Constant edge weights of the complete finite graphs ensure the convergence in all of their spanning subgraphs.

**Theorem** **3.** *In each arbitrary undirected simple finite connected graph G on n vertices with an arbitrary topology from the Markov chain, the convergence conditions (6)–(8) are certainly met when each edge is allocated the following weight (i.e., the mixing parameter of the Best Constant weights in the complete finite graph with the order n):*

(19) $$\epsilon =\frac{2}{\lambda_{1}\left(K_{n}\right)+\lambda_{n-1}\left(K_{n}\right)},$$

*and the diagonal elements of the weight matrix are set according to:*

(20) $${\left[W\left(k\right)\right]}_{ii}=1-\frac{2d_{i}\left(k\right)}{\lambda_{1}\left(K_{n}\right)+\lambda_{n-1}\left(K_{n}\right)},\;\mathrm{for}\;i\;=\;1,2,\;\ldots n$$

**Proof of Theorem** **3.** Each initial undirected simple finite connected graph on *n* vertices from the sequence forming a stationary Markov chain is a spanning subgraph of the complete finite graph *K*${}_{n}$ (Note that each complete finite graph is also a spanning subgraph of itself.) with an arbitrary number of removed edges. At first, let us recall that each undirected simple finite connected graph *G* on *n* vertices achieves the fastest convergence rate for the case when all of the edges take the same weight with the following mixing parameter ϵ [52]:

(21) $$\epsilon =\frac{2}{\lambda_{1}\left(G\right)+\lambda_{n-1}\left(G\right)}$$

Furthermore, let us determine the mixing parameter ϵ of the Best Constant weights in the complete finite graph *K*${}_{n}$, as follows:

(22) $$\epsilon =\frac{2}{\lambda_{1}\left(K_{n}\right)+\lambda_{n-1}\left(K_{n}\right)}$$

Based on (12) and (13) from Theorem 2, we can state:

(23) $$\lambda_{1}\left(G\right)\leq \lambda_{1}\left(K_{n}\right)\wedge \lambda_{n-1}\left(G\right)\leq \lambda_{n-1}\left(K_{n}\right)$$

Thus,

(24) $$\begin{array}{c} \lambda_{1}\left(G\right)\leq \lambda_{1}\left(K_{n}\right)\wedge \lambda_{n-1}\left(G\right)\leq \lambda_{n-1}\left(K_{n}\right)\Rightarrow \lambda_{1}\left(G\right)+\lambda_{n-1}\left(G\right)\leq \lambda_{1}\left(K_{n}\right)+\lambda_{n-1}\left(K_{n}\right)\Rightarrow \\ \frac{2}{\lambda_{1}\left(G\right)+\lambda_{n-1}\left(G\right)}\geq \frac{2}{\lambda_{1}\left(K_{n}\right)+\lambda_{n-1}\left(K_{n}\right)} \end{array}$$

According to [52], the upper and the lower bound of the mixing parameter ϵ in graph *G* are:

(25) $$0<\epsilon <\frac{2}{\lambda_{1}\left(G\right)}$$

As stated in [37], the second smallest eigenvalue $\lambda_{n-1}$(*G*) (sometimes referred to as the Fiedler eigenvalue) is greater than zero in each undirected simple finite connected graph:

(26) $$\lambda_{n-1}\left(G\right)>0$$

Thus, the following statement is valid in the undirected simple finite connected graphs:

(27) $$\lambda_{1}\left(G\right)\geq \lambda_{n-1}\left(G\right)\Rightarrow \lambda_{1}\left(G\right)>0$$

Applying (24) and (25), we can then state:

(28) $$\lambda_{1}\left(G\right)+\lambda_{n-1}\left(G\right)>\lambda_{n-1}\left(G\right)\Rightarrow 0<\frac{2}{\lambda_{1}\left(K_{n}\right)+\lambda_{n-1}\left(K_{n}\right)}\leq \frac{2}{\lambda_{1}\left(G\right)+\lambda_{n-1}\left(G\right)}<\frac{2}{\lambda_{1}\left(G\right)}$$

Accordingly, as seen above, $\frac{2}{\lambda_{1}\left(K_{n}\right)+\lambda_{n-1}\left(K_{n}\right)}$ is the upper bound ensuring that the convergence condition (8) is met in each spanning subgraph of *K*${}_{n}$. The values of the largest and the second smallest eigenvalue of the complete finite graph *K*${}_{n}$ can be easily determined by a mechanism for estimating eigenvalues or according to the graph order *n* determinable by various different techniques, such as AC, for graph order estimation, tagging, ordered numbering, etc. [19,53,54,55,56]. Now, it remains to define how the diagonal elements (i.e., the weight for the current inner state at a sensor node) have to be updated in order to meet the convergence conditions (6) and (7). These conditions are met, provided that the sum of all the entries in each row/column is equal to one. As each node *v*${}_{i}$ receives *d*${}_{i}$(*k*) messages at a *k*th iteration, the sum of the weights for all the received states can be determined, as follows:

(29) $$\epsilon d_{i}\left(k\right)$$

Hence, in order to meet the convergence conditions (6) and (7), the diagonal matrix entries have to take this value:

(30) $$1-\frac{2d_{i}\left(k\right)}{\lambda_{1}\left(K_{n}\right)+\lambda_{n-1}\left(K_{n}\right)}$$

Thus, the sum of all the entries in the weight matrix is equal to one, regardless of the values of both the degree of v${}_{i}$ and the Laplacian eigenvalues:

(31) $$1-\frac{2d_{i}\left(k\right)}{\lambda_{1}\left(K_{n}\right)+\lambda_{n-1}\left(K_{n}\right)}+\epsilon d_{i}\left(k\right)=1-\frac{2d_{i}\left(k\right)}{\lambda_{1}\left(K_{n}\right)+\lambda_{n-1}\left(K_{n}\right)}+\frac{2d_{i}\left(k\right)}{\lambda_{1}\left(K_{n}\right)+\lambda_{n-1}\left(K_{n}\right)}=1$$

Therefore, the following recalculation of the diagonal weights is fully-distributed (the sensor node have to be additionally aware only of their current degree *d*${}_{i}$(*k*)) and ensures that (6) and (7) are met:

(32) $${\left[W\left(k\right)\right]}_{ii}=1-\frac{2d_{i}\left(k\right)}{\lambda_{1}\left(K_{n}\right)+\lambda_{n-1}\left(K_{n}\right)},\;\mathrm{for}\;i\;=\;1,2,\;\ldots n$$

Thus, the weight matrix **W**(*k*) ensuring the convergence of AC in each arbitrary undirected simple finite graph from a Markov chain can be composed as the following doubly-stochastic matrix (A doubly-stochastic matrix is a matrix where the sum of all the entries in each row/collum is equal to one, and the entries of this matrix are non-negative.):

(33) $${\left[W\left(k\right)\right]}_{ij}=\left\{\begin{array}{cc} \frac{2}{\lambda_{1}\left(K_{n}\right)+\lambda_{n-1}\left(K_{n}\right)}, & \mathrm{if}\;e_{\mathrm{ij}}\in E\\ 1-\frac{2d_{i}\left(k\right)}{\lambda_{1}\left(K_{n}\right)+\lambda_{n-1}\left(K_{n}\right)}, & \mathrm{if}\;i=j\\ 0, & \mathrm{otherwise} \end{array}\right.$$

□

Thus, the only information that has to be determined before the algorithm begins and it is necessary for meeting the convergence conditions is the mixing parameter ϵ of the Best Constant weights in the complete finite graph on *n* vertices, and the only time-variant necessary information is that each entity *v*${}_{i}$ knows its current degree *d*${}_{i}$(*k*), which can be easily determined according to the number of the received messages at the corresponding iteration. Thus, the weights allocated to the edges do not have to be changed during the algorithm execution. As seen from (28), a drawback of our approach is that the mixing parameter ϵ can be lower than the Best Constant edge weights; therefore, the convergence rate can be decreased. Additionally, note that the mixing parameter ϵ can take also a lower value than the presented upper bound (22), however, the convergence rate is certainly decreased in this case.

### 4.3. Convergence Analysis in Critical Topologies

In what follows, we prove that the application of the weight matrix from (33) ensures the convergence also in undirected simple finite bipartite regular, undirected simple finite disconnected graphs, and undirected simple finite disconnected graphs whose component(s) are bipartite regular.

**Lemma** **1.** *Let G be an undirected simple finite bipartite regular connected graph on n vertices. Then, the weight matrix provided in (33) ensures the convergence of AC in this graph.*

**Proof of Lemma** **1.** At first, let us recall that [52]:

(34) $$\rho \left(W-\frac{1}{n}11^{T}\right)=max\left\{1-\epsilon \lambda_{n-1}\left(G\right),\epsilon \lambda_{1}\left(G\right)-1\right\}$$

Now, let us turn our attention to the upper bound of $\lambda_{1}$(*G*), which can be determined, as follows [57]:

(35) $$\lambda_{1}\left(G\right)\leq max\left\{\frac{\left(d_{i}+d_{j}\right)+\sqrt{{\left(d_{i}-d_{j}\right)}^{2}+4m_{i}m_{j}}}{2}:e_{ij}\in E\right\}$$

Here, *m*${}_{i}$ is the average of the degrees of the vertices that are adjacent to the corresponding vertex *v*${}_{i}$. As stated in [57], the equality holds in the bipartite regular graphs, and, therefore, the formula (35) can be simplified for these graphs, as follows:

(36) $$\lambda_{1}\left(G\right)=2\Delta$$

Here, Δ represents the maximum degree of a graph. Thus, applying (34) and (36), we obtain:

(37) $$\lambda_{1}\left(G\right)=2\Delta \Rightarrow \frac{1}{\Delta }2\Delta -1=1\Rightarrow \rho \left(W-\frac{1}{n}11^{T}\right)=1$$

Accordingly, we can see that the convergence condition (8) is broken for ϵ = $\frac{1}{\Delta }$. However, in the case of ϵ =$\frac{2}{\lambda_{1}\left(K_{n}\right)+\lambda_{n-1}\left(K_{n}\right)}$, the convergence is also ensured in undirected simple finite bipartite regular graphs, as:

(38) $$\begin{array}{c} \frac{2}{\lambda_{1}\left(K_{n}\right)+\lambda_{n-1}\left(K_{n}\right)}=\frac{1}{n}\wedge \Delta \leq n-1<n\Rightarrow \\ max\left\{1-\frac{2}{\lambda_{1}\left(K_{n}\right)+\lambda_{n-1}\left(K_{n}\right)}\lambda_{n-1}\left(G\right),\frac{2}{\lambda_{1}\left(K_{n}\right)+\lambda_{n-1}\left(K_{n}\right)}2\Delta -1\right\}<1\Rightarrow \\ \rho \left(W-\frac{1}{n}11^{T}\right)<1 \end{array}$$

□

**Figure 7:**
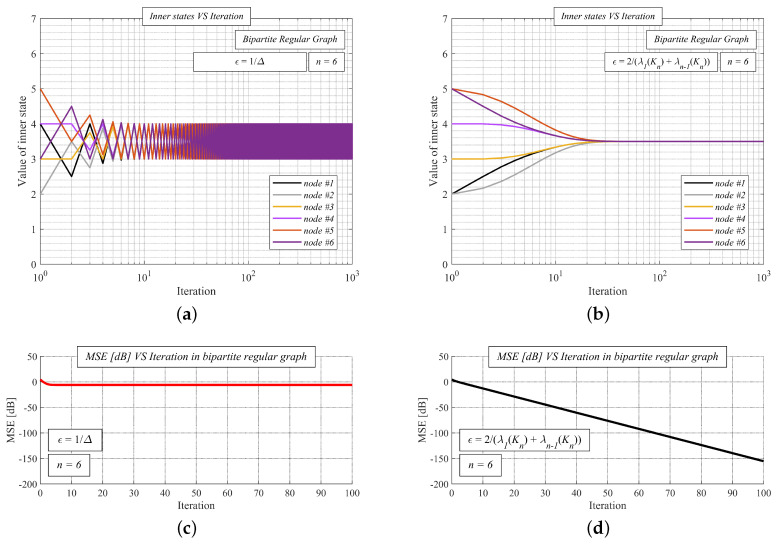
Comparison of average consensus with ϵ = $\frac{1}{\Delta }$ and ϵ = $\frac{2}{\lambda_{1}\left(K_{n}\right)+\lambda_{n-1}\left(K_{n}\right)}$ over random undirected simple finite bipartite regular graph. (**a**) Inner states VS iteration number–average consensus with ϵ =$\frac{1}{\Delta }$–algorithm diverges; (**b**) Inner states VS iteration number–average consensus with ϵ =$\frac{2}{\lambda_{1}\left(K_{n}\right)+\lambda_{n-1}\left(K_{n}\right)}$–algorithm converges; (**c**) Mean square error VS iteration number–average consensus with ϵ =$\frac{1}{\Delta }$—Mean square error does not decrease; (**d**) Mean square error VS iteration number–average consensus with ϵ =$\frac{2}{\lambda_{1}\left(K_{n}\right)+\lambda_{n-1}\left(K_{n}\right)}$—Mean square error decreases.

In Figure 7, we show the function of the inner states (see Figure 7a/Figure 7b) and MSE (see Figure 7c/Figure 7d) as the number of the iterations is increased over a random undirected simple finite bipartite regular graph with the order *n* = 6. We apply MSE over the iterations, a reasonable metric for performance evaluation defined, as follows [58]:

(39) $$\mathrm{MSE}\left(k\right)=\frac{1}{n}\sum_{i=1}^{n}{\left(x_{i}\left(k\right)-1^{T}\frac{x(0)}{n}\right)}^{2}$$

We analyze AC with two values of the mixing parameter ϵ - either ϵ = $\frac{1}{\Delta }$ or ϵ = $\frac{2}{\lambda_{1}\left(K_{n}\right)+\lambda_{n-1}\left(K_{n}\right)}$. The initial states are equal to the unique indexes (i.e., *x*${}_{1}$(0) = 1, *x*${}_{2}$(0) = 2, …). From Figure 7a, we can see that the mixing parameter ϵ = $\frac{1}{\Delta }$ does not ensure the correct functioning of the algorithm over the undirected simple finite bipartite regular graph, since the inner states do not converge to the arithmetic mean, meanwhile, ϵ = $\frac{2}{\lambda_{1}\left(K_{n}\right)+\lambda_{n-1}\left(K_{n}\right)}$ does (see Figure 7b). Regarding MSE, it can be seen that the error does not decrease, except for several iterations in the beginning in the case of ϵ = $\frac{1}{\Delta }$ (see Figure 7c). However, MSE is decreased as the iteration number increases for ϵ = $\frac{2}{\lambda_{1}\left(K_{n}\right)+\lambda_{n-1}\left(K_{n}\right)}$ (see Figure 7d)—thus, AC operates correctly in this case.

**Lemma** **2.** *Let G be an undirected simple finite disconnected graph on n vertices formed by components C${}_{1}$, C${}_{2}$,… (on m${}_{1}$, m${}_{2}$, … vertices). Subsequently, the weight matrix provided in (33) ensures the convergence of AC in this graph.*

**Proof of Lemma** **2.** As discussed in [17], the inner states converge to the arithmetic mean from all the initial states when the disconnected parts are merged during the algorithm execution. When *G* is disconnected and the convergence conditions are met, the inner states in each component *C*${}_{1}$, *C*${}_{2}$,… converge to the arithmetic mean determined by the average calculated from the inner states of all the vertices in a particular component (let us call it a local arithmetic mean) instead of to the arithmetic mean calculated from the inner states of all the vertices in *G* (let us call it the global arithmetic mean). The convergence is ensured for an arbitrary component *C*${}_{i}$, since (*i* is the index of an arbitrary component) ((28) is applied):

(40) $$\begin{array}{c} \frac{2}{\lambda_{1}\left(K_{n}\right)+\lambda_{n-1}\left(K_{n}\right)}=\frac{1}{n}\wedge m_{i}<n\Rightarrow \\ 0<\frac{2}{\lambda_{1}\left(K_{n}\right)+\lambda_{n-1}\left(K_{n}\right)}<\frac{1}{m_{i}}\leq \frac{2}{\lambda_{1}\left(C_{i}\right)+\lambda_{n-1}\left(C_{i}\right)}<\frac{2}{\lambda_{1}\left(C_{i}\right)} \end{array}$$

Thus, at the iterations when an arbitrary undirected simple finite graph is not connected, the inner states in its components converge to local arithmetic means; otherwise, they converge to the global arithmetic mean. In [36], the authors state that the consensus can be achieved in the case when the union of the graphs is connected frequently enough. □

**Figure 8:**
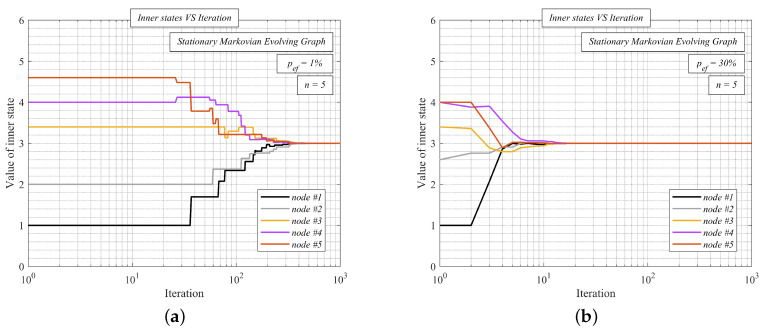
Inner states VS iteration number–average consensus with the proposed weight matrix over stationary Markovian evolving graph containing also disconnected graphs.

In Figure 8, we analyze AC with the presented upper bound (i.e., ϵ = $\frac{2}{\lambda_{1}\left(K_{n}\right)+\lambda_{n-1}\left(K_{n}\right)}$) over two randomly generated SMEGs with *p*${}_{ef}$ = 1% (see Figure 8a) and with *p*${}_{ef}$ = 30% (see Figure 8b)—like in the previous analysis, the initial inner states are equal to the unique indexes. From the presented results, we can see that the inner states converge to the arithmetic mean (equal to three) in both cases although the Markov chain contains also disconnected graphs. In Figure 8a, the inner states converge slower than in Figure 8b since connectivity is lower, and the graphs from a Markov chain are more frequently disconnected. Nevertheless, AC with the presented upper bounds operates correctly in both SMEGs.

**Lemma** **3.** *Let G be an undirected simple finite disconnected graph on n vertices, and let C be its arbitrary component on m vertices (m < n) that is bipartite and regular. Subsequently, the weight matrix provided in (33) ensures the convergence of AC.*

**Proof of Lemma** **3.** Analogically to Lemma 1, we can state that ϵ =$\frac{1}{\Delta^{C}}$ (Δ${}^{C}$ is the maximum degree of *C*) causes that the condition (8) is not met when *C* is bipartite regular. However, when ϵ = $\frac{2}{\lambda_{1}\left(K_{n}\right)+\lambda_{n-1}\left(K_{n}\right)}$, the convergence condition (8) is met in this bipartite regular component because:

(41) $$\begin{array}{c} \frac{2}{\lambda_{1}\left(K_{n}\right)+\lambda_{n-1}\left(K_{n}\right)}=\frac{1}{n}\wedge \Delta^{C}\leq m-1<m<n\Rightarrow \\ max\left\{1-\frac{2}{\lambda_{1}\left(K_{n}\right)+\lambda_{n-1}\left(K_{n}\right)}\lambda_{n-1}\left(C\right),\frac{2}{\lambda_{1}\left(K_{n}\right)+\lambda_{n-1}\left(K_{n}\right)}2\Delta^{C}-1\right\}<1\Rightarrow \\ \rho \left(W^{C}-\frac{1}{m}11^{T}\right)<1 \end{array}$$

Here, **W**${}^{C}$ is the weight matrix of component *C*. □

**Figure 9:**
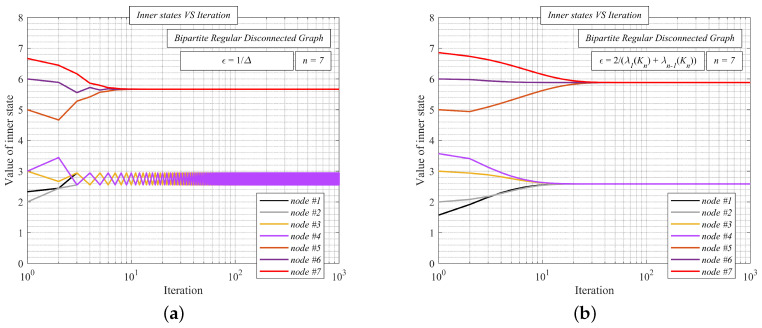
Inner states VS iteration number–average consensus with ϵ = $\frac{1}{\Delta }$ and ϵ = $\frac{2}{\lambda_{1}\left(K_{n}\right)+\lambda_{n-1}\left(K_{n}\right)}$ over random undirected simple finite disconnected graph with bipartite regular component.

Below, we show the function of the inner states over a randomly generated undirected simple finite disconnected graph formed by two components of which one is bipartite regular (this component is formed by the nodes #1, #2, #3, and #4). For performance evaluation, we apply MSE(*k*) (39). The initial inner states are equal to the unique indexes again. The mixing parameter ϵ takes two values: ϵ = $\frac{1}{\Delta }$ (Figure 9a) or ϵ = $\frac{2}{\lambda_{1}\left(K_{n}\right)+\lambda_{n-1}\left(K_{n}\right)}$ (Figure 9b). In Figure 9a, we can see that the inner states in one of the components do not converge to the local arithmetic mean as the convergence condition (8) is not met for ϵ = $\frac{1}{\Delta }$. However, our upper bound (i.e., ϵ = $\frac{2}{\lambda_{1}\left(K_{n}\right)+\lambda_{n-1}\left(K_{n}\right)}$) also ensures the convergence in the bipartite regular component, as shown in Figure 9b.

## 5. Experimental Section

### 5.1. Research Methodology

In this subsection, we introduce the research methodology applied in our experimental evaluations. All of the experimental evaluations presented in this paper are executed in Matlab2018a using both the authors’ software and built-in Matlab scripts. In our experimental evaluations, AC with the weight matrix (33) is tested over SMEGs and SEMEGs with a varied configuration. In SMEGs, the probability *p*${}_{ef}$ is changed from smaller values (representing networks with low connectivity) to larger ones (representing networks with high connectivity). Regarding SEMEGs, we analyze AC with the weight matrix (33) over three types of these graphs (labeled as SEMEGs I/SEMEGs II/SEMEGs III). In SEMEGs I, the birth-rate *p* and the death-rate *q* are equal to one another, meaning that a sensor node establishes a connection with another node as likely as this connection is terminated. In SEMEGs II, a connection is established with an unvaried probability; however, the probability of its termination is varied. In SEMEGs III, the probability of connection termination does not vary; meanwhile, the probability of connection establishment changes. In our experimental evaluations, we set the graph parameters to the following values, including also the critical topologies discussed above:
**SMEGs****SEMEGs I****SEMEGs II****SEMEGs III**•*p*${}_{ef}$ =   1 %•*p*${}_{ef}$ =   5 %•*p*${}_{ef}$ = 10 %•*p*${}_{ef}$ = 20 %•*p*${}_{ef}$ = 30 %•*p* =   1 %, *q* =   1 %•*p* =   5 %, *q* =   5 %•*p* = 10 %, *q* = 10 %•*p* = 20 %, *q* = 20 %•*p* = 30 %, *q* = 30 %•*p* = 1 %, *q* =   1 %•*p* = 1 %, *q* =   5 %•*p* = 1 %, *q* = 10 %•*p* = 1 %, *q* = 20 %•*p* = 1 %, *q* = 30 %•*p* =   1 %, *q* = 30 %•*p* =   5 %, *q* = 30 %•*p* = 10 %, *q* = 30 %•*p* = 20 %, *q* = 30 %•*p* = 30 %, *q* = 30 %


The performance analysis that is presented in this paper consists of three different numerical evaluations; therefore, the following subsection is divided into three parts as follows:

• Part I.—MSE is evaluated during the first 100 iterations over SMEGs and during the first 50 iterations over SEMEGs. For all the graph configurations in each scenario, 100 unique graphs formed by 200 vertices (i.e., *n* = 200) are generated. In the presented figures, MSE averaged over 100 SMEGs/SEMEGs for all the graph configurations is depicted and furthermore analyzed.
• Part II.—The numerical values of the inner states as a function of the iteration number are analyzed over the first 1000 iterations in order to show how the inner states evolve. In each figure, the results over one graph are depicted. In order to ensure good readability of the paper, the functions for only some graph configurations are provided.
• Part III.—The convergence rate expressed as the number of the iterations for the consensus achievement is shown for all of the graph configuration in each analyzed scenario. For all of the graph configurations in each scenario, 100 unique graphs formed by 200 vertices are generated like in Part I. In the case of AC, the inner states asymptotically converge to the arithmetic mean; therefore, it is necessary to apply a stopping criterion to bound the execution of AC. In our analyses, we apply the following one:
  (42) ∣max{x(k)}−min{x(k)}∣<P
  A lower value of P ensures a higher precision of the final estimates, but at the cost of a deceleration of the algorithm. We set the value of this parameter to 0.0001.

**Figure 10:**
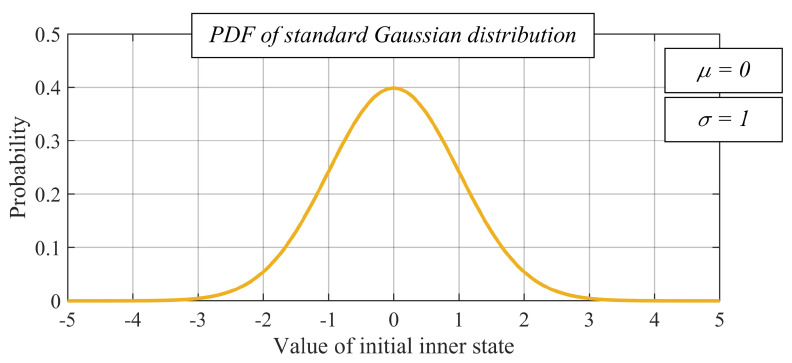
Probability density function of standard Gaussian distribution.

Regarding the initial states, they are independent and identically distributed random values of the standard Gaussian distribution (see Figure 10) in all of the experimental evaluations, i.e.,:

(43) $$x_{i}\left(0\right)∼N\left(0,1\right),\;for\;\forall v_{i}\in V$$

### 5.2. Performance Analysis over Stationary Markovian Evolving Graphs/Stationary Edge-Markovian Evolving Graphs

**Figure 11:**
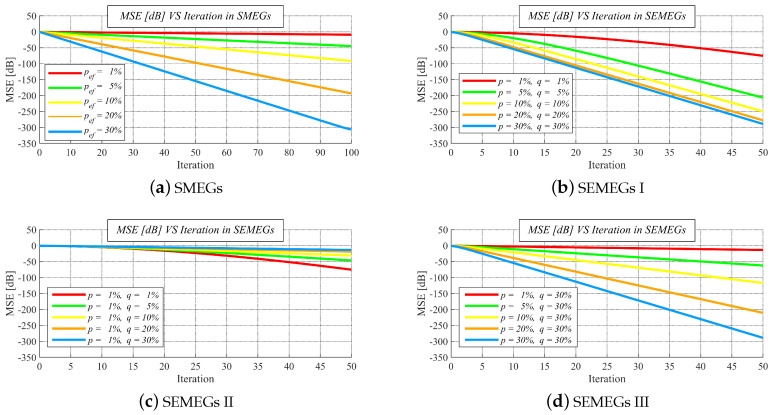
Performance of average consensus with designed weight matrix over stationary Markovian evolving graphs and stationary edge-Markovian evolving graphs with varied parameters.

As already mentioned, we analyze MSE over the iterations in Part I. From the results shown in Figure 11a–d, we can see that an increase in the iteration number ensures a decrease in MSE for each *p*${}_{ef}$ and for each pair *p*, *q*. In SMEGs, it can be observed that even also for *p*${}_{ef}$ = 1%, when the most graphs from Markov chains are disconnected, MSE slightly decreases. Additionally, it is observed that the algorithm achieves higher performance in graphs of higher connectivity (ensured by an increase in *p* in SMEGs, an increase in both *p*, *q* in SEMEGs I, a decrease in *q* in SEMEGs II, and an increase in *p* in SEMEGs III) than in the less connected ones. So, the most important fact demonstrated by these results is that the convergence is ensured over various mobile systems when the matrix that is provided in (33) is applied.

**Figure 12:**
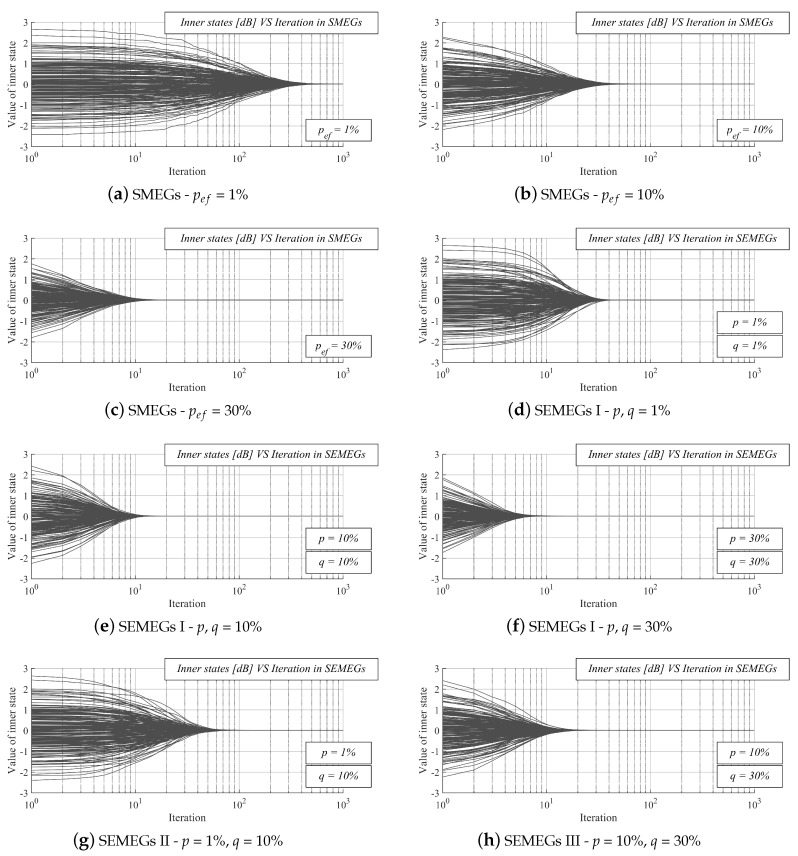
Evolution of inner states in the case of applying the weight matrix (33).

In Part II, we turn our attention to the graphs where the inner states as a function of the iteration number are analyzed (see Figure 12). In the graphs, the dark gray solid lines represent the inner states of all the sensor nodes (thus, 200 lines are shown in each graph). From the results, it can be seen that all if the inner states converge to the one value (equal to the arithmetic mean), proving that the algorithm operates correctly with the weight matrix (33). In Figure 12a–c and Figure 12d–f, an increase in *p*${}_{ef}$ and both *p*, *q* causes that the inner states faster approach the arithmetic mean. Additionally, in the example from Scenario 3 (see Figure 12g) and Scenario 4 (see Figure 12h), the inner states converge to the value of the arithmetic mean. Thus, the presented figures are another valid example that our contribution ensures that AC operates properly.

**Figure 13:**
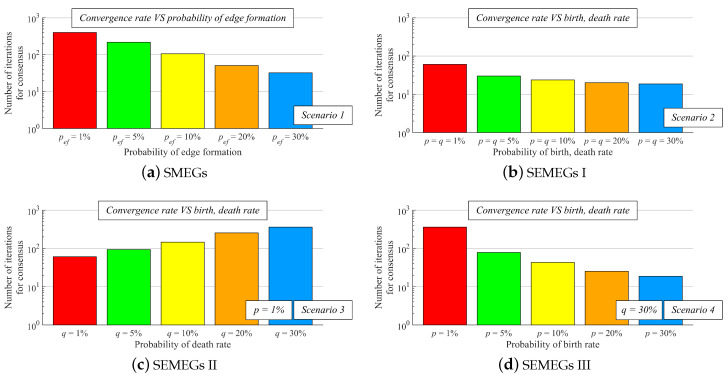
Convergence rate expressed as number of iteration for consensus achievement in the case of applying the weight matrix (33).

In Part III, we analyze the convergence rate of our contribution represented as the number of iteration for the consensus achievement—the stopping criterion (42) is applied to bound the execution of AC. From the results shown in Figure 13, it can be observed that an increase in *p*${}_{ef}$ in Scenario 1, both *p*, *q* in Scenario 2, and *p* in Scenario 4 ensures a decrease in the iteration number for the consensus achievement, i.e., the convergence rate is optimized as connectivity is increased. In Scenario 3, the convergence rate decreases as the value of *q* is increased. Again, it is proven that AC with the weight matrix (33) operates correctly, since the stopping criterion (42) is met in each graph.

In the last paragraph, we turn our attention to the drawbacks of our solution. It is not optimal in scenarios when the position of the sensor nodes is changed very slowly, i.e., the graph topology does not change in spite of the mobility or the topology changes so rarely that the parameters, such as the maximum degree, the Laplacian spectrum, etc., can be effectively determined. Additionally, our solution may not be appropriate in the case when one sensor nodes (or a small number) is mobile, meanwhile, the others are static—in this situation, it is not difficult to track and to reconfigure parameters such as the maximum degree, the degrees of the neighbors, etc. Furthermore, our approach is not supposed to be applied in static networks as the convergence rate can be decreased.

## 6. Conclusions

The research presented in this paper is motivated by an effort to simplify AC over MWSNs whereby the energy requirements are optimized, and the proper operation of the algorithm is ensured. We propose a weight matrix simplifying AC over MWSNs in communication and computation demands. Our contribution is based on the findings that the mixing parameter of Best Constant weights of the complete finite graph with an arbitrary order results in the convergence in all of its spanning subgraph without any edge reconfiguration. Our outcome is a weight matrix that guarantees the convergence over time-varying topologies, including critical topologies, such as disconnected and bipartite regular. Several numerical evaluations are carried out in order to demonstrate that the presented weight matrix ensures the convergence of AC over various mobile systems.

## Abbreviations

The following abbreviations are used in this manuscript:

AC	Average consensus algorithm
IoT	Internet of Things
MSE	Mean square error
MWSN	Mobile wireless sensor network
PDF	Probability density function
SEMEG	Stationary edge-Markovian evolving graph
SMEG	Stationary Markovian evolving graph
WSN	Wireless sensor network
